# Experiences of students with chronic illness in university education in Ireland

**DOI:** 10.1177/17423953241282246

**Published:** 2024-09-05

**Authors:** Olga Doris, Eimear C Morrissey

**Affiliations:** School of Psychology, 8799 University of Galway, Galway, Ireland

**Keywords:** Students, university education, chronic illness, qualitative

## Abstract

**Objective:**

The aim of this study was to explore the experiences of university students with a chronic illness in Ireland. The study also aimed to gain insight into students’ experiences with Disability Support Services (DSS) and identify gaps where additional supports and resources are needed.

**Design:**

Cross-sectional qualitative study.

**Methods:**

Fourteen students from three Irish universities participated in semi-structured interviews. The interviews were audio-recorded, transcribed, and analysed through the six-step process of reflexive thematic analysis.

**Results:**

Four themes were developed: (1) The burden of managing a chronic illness alongside university education; (2) Interruptions, disruptions and alterations to college life; (3) Flexible supports for fluctuating conditions; (4) Achieving in educating while living with a chronic illness.

**Conclusions:**

Participants reported a physical and emotional burden. Despite engaging in rigorous management strategies, many participants missed lectures and socialising with peers. Some found the supports from DSS to be useful, however many were unsure if they qualified for support, or found the supports available to be generic and inadequate for their needs. There is significant scope for the delivery of both teaching and DSS to be improved for this cohort, ensuring that all students, regardless of their health status, have equal opportunities for success.

## Introduction

The prevalence of chronic illnesses is growing worldwide^1^ and particularly so in Ireland where 31% of adults are now reported to be living with a long term condition.^2^ Concurrently, the rate of university education in Ireland has also increased, with 236,750 students in university education in 2022/2023,^3^ representing 4.5% of the total population of Ireland. Chronic illness can represent a significant challenge to successful undertaking of university education, as students need to successfully juggle the requirements of study with the ongoing management of their condition.

In Irish Law, chronic illness has been recognised as a category of disability under the Equal Status Act (2000). Educational institutions in Ireland, therefore, are legally obligated to provide reasonable accommodations to ensure students with chronic illnesses have equal opportunity to succeed. Supports are offered in most universities in Ireland through Disability Support Services (DSS), however international research has indicated that these types of supports are often inadequate for the unpredictable and fluctuating nature of chronic illness^4,5^ and are often set up for more predictable and stable concepts of disability.^6,7^ Accordingly, studies have shown that these students are less likely to disclose or seek supports compared to students with other types of disability.^8–10^

Without support services to meet individual needs, students may struggle with balancing the demands of a chronic illness with the expectations and tasks of university education, leading some students to experience psychological distress and self-exclude from aspects of college life. In a cross-sectional study on college students with and without chronic illness, Coutinho et al. (2019) found that students living with chronic illness had higher levels of anxiety and experiential avoidance and lower levels of quality of life.^11^ In a similar study, Johnston et al. (2023) found that students with a chronic illness had greater symptoms of anxiety and depression, more stressful life events and lower social support.^12^ Qualitative research has described how symptoms and impairments can negatively impact on studying and socialising^5,13^ and one US study has indicated that students with chronic illnesses are less likely to graduate college.^14^ The campus environment and supports available appear to be a key factor in this, with a two-wave study by Shrout and Weigel (2022) showing that stigma, alienation and lack of campus fit were associated with great illness-related academic interference, negative academic self-comparison, academic anxiety, academic dissatisfaction and lower expected grades.^15^ Hamilton et al. (2020) further developed this with an open-ended survey, finding that students perceived many staff members as lacking understanding and ‘policing’ academic regulations rather than providing support and accommodations.

Despite the growing importance of the issue, there is a paucity of research on the lived experience of students with chronic illnesses in university, and particularly so in Ireland, where research to date has focused on general disability (e.g.,^16,17^) or specific conditions (e.g.,^18^). Investigation into both the experience and the support needs of this group is needed to inform evidence-based policies, practices and interventions that foster inclusivity and equity. The aim of this study was to explore the experiences of students with a chronic illness in university education in Ireland. The study also aimed to gain insight into students’ experiences with DSS and identify areas where additional supports and resources may be warranted.

## Methods

### Design

This was a cross-sectional qualitative study, using individual semi-structured interviews. A qualitative descriptive approach as described by Sandelowski^19,20^ was used, as it allowed the production of a “data-near” and unadorned description of the experiences of participants. This study is reported in accordance with the Consolidated Criteria for Reporting Qualitative Research (see supplementary information).^21^ Ethical approval was granted by the University of Galway School of Psychology Research Ethics Committee.

### Participants

Participants were students in universities in Ireland who self-identified as having a chronic illness. Purposive sampling, incorporating convenience and snowballing strategies, were used to recruit participants. Eligible participants were invited to participate via advertisement which was placed on social media and emailed to chronic illness Non-Governmental Organisations (e.g., Diabetes Ireland, Arthritis Ireland, etc.) for placement on their social media networks. Participants were also asked to share the invitation with any chronic illness support groups they were members of, and with other eligible students. The study was also advertised in the Students’ Union weekly newsletter at the researchers’ university. Sample size was determined by the researcher, based on the concept of information power,^22^ the aim of the study, the analytic approach and previous research in the area e.g.,^6,23^ With these factors in mind, we aimed to recruit between 12–15 participants.

### Procedure

Following advertisement of the study, students who contacted the researcher to express their interest in participating in the research were emailed the participant information sheet. If willing to proceed, they were sent the online consent form and demographic questionnaire, following which the interview was arranged, to take place over Microsoft Teams. Consent was revisited at the beginning of the interview and all participants gave additional verbal consent. Interviews took place from February 2023 to May 2023. They were semi-structured and followed an interview guide (see supplementary information), developed by research team following the guidance of Braun & Clarke^24^ and informed by existing literature. All interviews were conducted by OD, a female non-clinical researcher with a background in health psychology. Interviews were transcribed verbatim with identifying details removed and names pseudonymised. Interviews ranged in length from 25 min to 74 min (*M *= 39 min). NVivo 12 was used to manage data.

### Data analysis

Data analysis was conducted by OD and followed the six step process of reflexive thematic analysis; familiarisation with the data, generating initial codes, generating themes, reviewing potential themes, defining and naming themes and producing the report.^24–26^ Reflexive thematic analysis was chosen as it allows an experiential analysis of patterned meaning that can give voice to the participants experiences. Transcripts were coded using an inductive data-derived approach. Codes were reviewed to identify patterns in the data and organised into clusters from which candidate themes and sub-themes were identified. Candidate themes and sub-themes were reviewed and revised in a recursive process until final themes were developed. All initial coding and theme generation was conducted by OD. EM (a female health psychologist and supervisor of OD) served as a ‘critical friend,’ providing peer debriefing by challenging and developing OD's interpretations. This process involved critical analysis of the coding and themes, ensuring the dependability of the findings. Due to time and resource constraints, transcripts and coding were not returned to participants for member checking.

## Results

Fourteen students from three universities in Ireland took part. All participant pseudonyms and characteristics can be seen in Table 1.

**Table 1.** Participant pseudonyms and characteristics.

Pseudonym	Age	Gender	Reported Chronic Illness(es)	Level of Study	Year and Mode of Study	Registered for Disability Support Services
Emma	33	Woman	Fibromyalgia; psoriatic arthritis	Postgraduate (Masters)	1^st^ (part-time)	In process of registering
John	26	Man	Type 1 diabetes	Undergraduate	1^st^ (part-time)	Yes
Sarah	26	Woman	Myalgic encephalomyelitis/chronic fatigue syndrome (ME/CFS)	Postgraduate (Masters)	2^nd^ /final (full-time)	Yes
Ciara	22	Woman	Type 1 diabetes	Undergraduate	4^th^ /final (full-time)	Yes
Sophie	23	Non-binary	Fibromyalgia; migraine, polycystic ovary syndrome; sciatica; tinnitus	Undergraduate	4^th^ /final (full-time)	Yes
Orla	20	Woman	Type 1 diabetes	Undergraduate	2^nd^ (full-time)	No
Niamh	20	Woman	Chronic pain	Undergraduate	3^rd^ (full-time)	No
Aoife	21	Woman	Postural orthostatic tachycardia syndrome (POTS)	Undergraduate	1^st^ (full-time)	Yes
Shane	20	Man	Ulcerative colitis	Undergraduate	2^nd^ (full-time)	Yes
Ava	19	Woman	Sickle cell anaemia	Undergraduate	2^nd^ (full-time)	Yes
Erin	26	Woman	Endometriosis; psoriasis; asthma	Postgraduate (PhD)	3^rd^ (full-time)	No
Michael	32	Man	Psoriasis; psoriatic arthritis; asthma	Postgraduate (Masters)	2^nd^ /final year (full-time)	Yes
Robert	19	Man	Crohn's disease	Undergraduate	2^nd^ year (full-time)	Yes
Tom	28	Man	Multiple sclerosis (MS)	Postgraduate (Masters)	2^nd^ /final year (full-time)	No

Four themes with corresponding subthemes were developed (see Table 2) and are described in detail below.

**Table 2.** Themes and subthemes generated through reflexive thematic analysis.

Themes	Subthemes
1. “There's just more things to worry about”: The burden of managing a chronic illness alongside university education	1.1 Time spent planning and organising
	1.2 Navigating support systems: An added burden
	1.3 Emotional burden
2. “It has affected my day-to-day life… study, work and social”: Interruptions, disruptions and alterations to college life	2.1 Drop-out and delays
	2.2 Daily disruptions
	2.3 Missing out on the social side
3. “It's kind of one-size-fits-all currently and there needs to be just more fluidity within that”: Flexible supports for fluctuating conditions	3.1 Navigating the fluctuating nature of chronic illnesses
	3.2 A need for personalised and flexible systems
4. “We are capable of making it academically, we just need some support”: Achieving in education while living with a chronic illness	4.1 Effective support systems and accommodations
	4.2 Peer and social support networks
	4.3 Staff support: Mixed experiences

### Theme 1: “There's just more things to worry about”: The burden of managing a chronic illness alongside university education

This theme centres on the burden of living with and managing a chronic condition while also trying to balance the workload and social expectations of university education.

#### Subtheme 1.1: Time spent planning and organising

Many participants described spending a lot of time planning and organising routines that allowed them to function in university. This level of planning was often required to mitigate for flare-ups and avoid worsening of symptoms. Michael described “*try[ing] to get ahead of everything*” and “*hav[ing] them* [assignments] *ready to submit relatively early, like a week or so out from the deadline*”. As well as assignments, participants spoke about spending time planning daily tasks. Niamh illustrated this, saying “*the night before I’ll plan my day, so I know I am not gonna be stuck walking loads*”. She found this helpful as the idea of an unplanned walk to a lecture or lab made her feel “*panicky*”. Ciara, on the other hand, described times when she was not organised, and her diabetes management tasks resulted in her being late to class or social events. She found reminding herself that other students do not have this extra burden to be useful.

“I don’t know any other type 1 s [diabetes] in college. So, when I am comparing myself and feeling like I’m not doing enough or I’m not performing well enough, I try to take it into account that there is all this management that other people don’t have to do.” (Ciara)

All of this planning and organisation could take a mental toll, with Robert summarising it as “*there's just more things to worry about*”.

#### Subtheme 1.2: Navigating support systems: An added burden

On top of the extra tasks involved in planning and organising, participants found that the process of accessing DSS support could add to the difficulties they were experiencing. Communication with teaching staff and DSS seemed to be a challenge in a lot of universities. Aoife talked about the process of getting an exemption from class due to a doctor's appointment.

“I’m probably gonna have to e-mail, like, three or four different people and then e-mail back and forth from my doctor asking for a letter and then they’ll say that letter is not substantial, so I have to get a different letter, but I already have a diagnosed chronic illness that the disability services are aware of.” (Aoife)

Ciara shared the experience of having to send a lot of emails to access an exam accommodation, describing it as “*a lot of stress and time*”. The poor standard of communication from DSS had quite negative consequences in some cases, as Orla had tried to register by “*contacting their e-mail but I haven’t gotten any reply*” which had led her not receiving any support.

#### Subtheme 1.3: Emotional burden

Managing a chronic condition in university was quite a heavy emotional burden for some participants. A lot of participants worried that they might be perceived as lazy by their peers and lecturers.

“People don’t realise that other people don’t have perfect health. It’s, like, oh, this person’s always skipping college, but, like, it’s not the case. They could be at home with a migraine or something, but they’re still able to catch up academically …, even lecturers, I’ve noticed some of them just don’t understand, that’s our chronic illness, it’s not always laziness” (Aoife)

Aoife also described internalising this perception and “*always struggl[ing] with it, always being ashamed of it, thinking I’m lazy*”. This was shared by Niamh who felt “*guilty and lazy*” for getting the bus to classes when her peers were walking.

This fear of judgement extended to other symptoms too. Michael, who has a visible skin condition, described how it made him “*concerned about how you present yourself to the world, which can impact where you go or who you hang out with for study reasons*” and Erin described an incident where a male student “*gave out to me for using a hot water bottle*” for cramps due to her endometriosis. John was “*always a bit angsty*” about his continuous glucose alarm beeping during class.

Worries about symptom management were common across participants too. Shane was “*scared of having to run to the toilet during the exam*” and Ava described having “*to leave class and …. just call my mom*” when she got a flare up of sickle cell anaemia symptoms and described “*the stress of having the pain during the exam*”. Robert described how a lack of clarity around DSS accommodations could also cause apprehension, saying he would “*be worried about how the exams and the different venue would work.*”

### Theme 2: “It has affected my day-to-day life… study, work and social”: Interruptions, disruptions and alterations to college life

This theme describes how chronic illnesses can interrupt and disrupt functioning in university education.

#### Subtheme 2.1: Drop-out and delays

Several of the participants had their university education either significantly delayed or disrupted by their condition. Both Emma and John had previously left courses.

“I tried full time college and then, just…, it started to impact on my mental health, with the diabetes on top of that, just the constant mood changes and stuff like that just…, ended up, the college didn’t work out for me.” (John)

A relapse in symptoms for Sarah meant that she *“had to go off books for the year because I was just so unwell*”.

Michael felt he was unable to start university education until he had “*assistance from the health service*” and “*proper medication*” which meant he was a mature student by the time he started. He also felt that the lack of information about available supports was problematic.

“If it was better known for students who were suffering illness there, that, like, there are supports for you because I didn’t think there’d be any supports for me, and I spent nine years not doing the third level [university].” (Michael)

#### Subtheme 2.2: Daily disruptions

Flare-ups of symptoms could cause significant disruption to the daily functioning of all participants. Problems with concentration were frequently mentioned, with Sarah saying “*Academically I*’*d be very much so at the mercy of getting brain fog*”. Michael described flare-ups of pain which “*make it very difficult to sleep properly …, which then impacts my ability to concentrate or my motivation to stay in the library all day, for example*”, which was similar to Emma, who “*would have flare ups of pain and fatigue and it would affect my cognitive abilities also*”.

Ava shared how constant background monitoring for flare-ups could also have an impact.

“My attention span would also be another thing, just because I’m more, just conscious of, because I get, like, warning signals, if I’m about to get a flare up and I’d just be more conscious of that than listening in the lecture” (Ava)

John found that “*trying to get the motivation to actually sit down and do stuff when your blood sugar's out, kind of out of control, it can be tough,*”. Managing high and low blood glucose levels was a problem also shared by Orla and Ciara. Orla outlined how it could impact her assignment performance.

“So if I’m in the middle of a lab and all of a sudden I feel sick, or I feel like I’m having low blood sugar, then I have to step out…and then when I’m going to write my report or do my assignment for whatever the lab was, I can feel that I’m at a bit of a disadvantage because I haven’t been able to sit through the entire lab”.

Falling behind in class could then trigger a stress response, which in turn triggered more flare-ups. Ciara described this cycle as “*you*’*re really stressed about something, and then you also have to deal with having high blood sugar, which is like being sick on top of it, which that in turn, makes you stressed*”.

#### Subtheme 2.3: Missing out on the social side

Many participants described limits to the social side of their university experience. Ava stated that “*my social life is different to everyone elses*” as she is “*very restricted in what I can do*”. She described how fear of a sickle cell crisis meant that she “*can*’*t really participate in some activities with my friends, like going out and stuff*”. Ciara, on the other hand, tried to prioritise social activity but found it be difficult as “*there*’*s so much that takes up my time with diabetes*” and it can be “*exhausting*”.

Some participants talked about how they need to prioritise academic work over social activities. Aoife often “*miss[ed] out*” on socialising with her friends because she attended all her lectures and felt “*terrible*” by the end of the day. Sarah had to “*assess what*’*s a priority*” and had to “*cancel things socially, so I can make up for my academics*”. Emma, who as mentioned, had to previously drop out of a course due to her illness was so “*determined to get through academically*” that “*socialising was just taken out of the equation*”. She acknowledged that this focus mean that she was missing out on the “*college experience*”.

### Theme 3: “It’s kind of one-size-fits-all currently and there needs to be just more fluidity within that”: Flexible supports for fluctuating conditions

The theme encapsulates participants’ experiences of the challenges and support needs associated with their chronic illnesses.

#### Subtheme 3.1: Navigating the fluctuating nature of chronic conditions

All participants emphasised the need for flexible supports for those living with chronic illnesses. This is due to the fluctuating and often invisible nature of their conditions.

“it would be helpful for people to understand that… chronic illness fluctuates over time and maybe one day you look perfectly fine and you can do ten million things and more, and then the next day, you’re just incapacitated and you’re lying in bed”. (Emma)

Tom had a keen sense of imposter syndrome because “*I feel quite healthy most of the time but it, it kind of comes in like waves*”. This made him doubt his eligibility for DSS saying,

“that kind of would have been a thing that was on my mind in terms of accessing supports, that, I wasn’t really sure if I deserve them…” (Tom)

Sophie felt this perception in other people.

“It’s the same way as if someone’s in a wheelchair, they need a ramp to get in the door. That is fine, people have accepted that, whereas they’re not accepting, you look fine, so you must be fine, type thing.”

However, she emphasised that this did not mean that invisible conditions were any less deserving of support than visible conditions.

#### Subtheme 3.2: A need for personalised and flexible systems

Many participants felt that the current method of delivering teaching was not set up to meet their needs. Aoife was often unable to attend classes in-person but caught up on content online. She felt that in this context, grading for in-person attendance was very unfair.

“And then things like some lectures are mandatory, to be in person and if I miss that I’m gonna lose part of my grade. And I’m like, I can’t, but I’m still learning the same content as everyone else”.

Some of the participants had been in university education during the COVID-19 pandemic when classes moved online. This made the experience much easier for Sarah who said, “*well actually the fact that we went into a pandemic and everything went online, the masters actually suited me, to be at home, not commuting*”. Emma and John were both currently doing part-time courses with mainly online delivery, and both found that it suited their needs well.

Shane and Aoife both felt quite angry that the option of online delivery or recorded lectures was taken away after the pandemic and felt it was quite unfair. Shane said “*Last year was grand because all the lectures were recorded but…whereas, this year, now…if I am sick there*’*s not much I can do. I can actually look at the notes but most of the stuff said, it will be in the lectures*.” He also claimed that in this context, he was essentially “*being punished for being sick”.*

There was also a feeling that DSS could be improved with a more personalised and flexible offering. Ciara described not receiving some requested accommodations because her advisor “*had other type 1 s [diabetes] under her advisement who did not need these accommodations, so, why was I asking for them*”. Sophie also would have liked a more tailored approach.

“They have a bunch of services, but if they’re not taking the time to explain the services or asking you about yourself and stuff in depth, they’re not going to know what services you might need and then what ones can apply to you.” (Sophie)

### Theme 4: “We are capable of making it academically, we just need some support”: Achieving in education while living with a chronic illness

#### Subtheme 4.1: Effective support systems and accommodations

Many of the participants shared positive experiences they had had during their time in university. DSS accommodations had made a big difference to several participants. Michael described a useful assistive technology he had been provided with, saying

“it’s a speech to text and text to speech software, in case my hands were acting up someday, that ended up being very helpful” (Michael)

Exam accommodations were also viewed very positively.

“I was able to sit my exams in a small room, instead of sitting in with thousands of people. That saved a lot of travel and a lot of stress and, it, it just limited the kind of sensory information I was getting as well, so that again helped get me through the exams.” (Emma)

Sophie was able to avail of a respite room “*where I can just, go and take a nap during the day, which has been really helpful*”. This would have been a useful resource in Aoife’s university, as she described long gaps between lectures and “*there is nowhere to go. So, I’m kind of walking around most the day, and, by the end, I get a migraine type of thing.”*

Simply “*being believed*” was seen as a huge support by many participants.

“That’s the kind of attitude that I found across the department, is really great, you know, just that understanding, the willingness to be flexible, even if they don’t know what diabetes is like. They just understand that look, this is a chronic illness, we believe her, you know, it’s really great.” (Ciara)

This was often aided by being registered with DSS as Sarah described “*just, being registered with them helped…it kind of helped lecturers understand that there really was an issue*”.

#### Subtheme 4.2: Peer and social support networks

While none of the participants were actively engaged in a peer support group, most were very enthusiastic about the concept.

“I think hearing about other people’s experiences, like, similar experience could help me feel better.” (Ava)

“if there was a way that I could get in touch with somebody, like even a third year or a fourth year, it’d be really helpful because, they’re in the same experience as you, so they might have dealt with something that they can teach you and you might have dealt with something that you can help them with” (Orla)

Some participants had received support from their classmates and friends. Emma described how classmates helped her out, “*I missed a lot of classes, so I was always asking for notes from people, they would help me, you know, share class notes and get me up to date*”. Shane kept his friends up to date with his condition because “*it*’*s nice for people just to know exactly what it is as well, just…because they can help me if I need help*.”

#### Subtheme 4.3: Staff support: Mixed experiences

Participants had mixed experiences of support from teaching staff. Sometimes there was a feeling that lecturers didn’t fully understand support needs.

“I was doing the exam from home but I still needed accommodation such as 10 min after reading time, and, like, if my blood sugars went low, I need to be able to stop and address that and he just kept saying ‘‘no, you’re taking it from the comfort of your own home, you shouldn’t have any problems’’, and he just didn’t grasp that disability doesn’t park itself at your front door, you know, it comes into the room with you at all times”. (Ciara)

However, many participants had positive experiences with teaching staff and felt that this helped them to achieve.

“I was able to confide in them, explain the difficulties I was having and once I had that diagnosis, I was able to show them a piece of paper … that helped me get extensions which really helped me get through my program.” (Emma)

Sarah had similar support, saying “*they always were there to cater to whatever it was I needed, usually more time*”. This kind of support from teaching staff, along with support from DSS, felt truly enabling for some.

“Just for anyone who thinks that, their illness…will necessarily hold them back…it’s, it’s not the case, like, we might need extra supports, but we can do just as much as anyone else, you know.” (Michael)

## Discussion

This study explored the experiences of students with a chronic illness in university education in Ireland. The themes generated underscore the significant challenges that this cohort face. *The burden of managing a chronic illness alongside university education* (theme 1) can be both physically and emotionally taxing. Many participants engaged in rigorous organisation, time management and prioritisation strategies to overcome these challenges, but still often felt at the mercy of their symptoms and the stress-flare-up cycle. A fear of judgement and stigma was expressed by many participants, and this extended to both their peers and teaching staff. *Interruptions, disruptions, and alterations to college life* (theme 2) were common and in-person lectures and socialising activities were often missed out on, leading to feelings of isolation and frustration.

The need for *flexible supports for fluctuating conditions* (theme 3) was evident. Over half of the participants had registered with DSS within their university, and many had found supports such as equipment and extra time for exams useful. However, due to the fluctuating and sometimes invisible natures of their conditions, many of the participants found the supports available to be somewhat generic and inadequate for their needs. Participants who had not registered for DSS cited uncertainty around their eligibility and were unsure whether their illness would be considered a disability. This is in contrast to previous findings^5,27^ where disclosure was withheld due to an outright rejection of the disabled label. DSS was often seen to have a conceptualisation of disability as stable and predictable, and participants felt that the system of support was not set up to meet their fluctuating and varying support needs. A particular frustration for many students was the lack of online learning options, a mode of delivery that ensured their inclusion in class during a flare-up of symptoms or hospital visits. This was particularly felt by those who had received online teaching during the COVID-19 pandemic, and subsequently had it removed. One student described it as “*just being punished for being sick*”, a phrase that articulates the gap between legal requirement of equal opportunities for success, and what is happening in practice.

Participants gave many examples of managing well and receiving appropriate support, demonstrating that it is possible to have a campus environment that meets the requirements of students living with chronic illness. This *achievement while living with a chronic illness* (theme 4) was facilitated by several factors. The continuation of recorded lectures was appreciated by those who had access to them, implying that teaching, where possible, should be delivered in a hybrid manner. While none of the participants were actively engaged in a peer support group, most spoke very positively about the idea of having one on campus. If implemented well, this type of support could provide a sense of belonging and understanding, and offer a valuable complement to DSS.^28^ Often just “*being believed*” by teaching staff offered a validation that was reassured students that they would receive appropriate accommodations and avoid judgement. Education for both DSS and teaching staff on nature of chronic illness and the need for anticipatory adjustments, as well as their legal basis, would inform an inclusive approach to teaching and assessment.^4^ Additionally, having a dedicated full-time DSS advisor for students with chronic illnesses could result in a more holistic support service for students.^6^ Participants with a consistent point of contact who advocated and navigated with institutional systems on their behalf seemed to have a much more positive experience than those that did not. While some of the participants did have a dedicated advisor, many found them difficult to contact as they were working part-time, leading to a sense of being under-prioritised and under-valued, a finding reflected in Hamilton et al. (2021).^4^ A tailored and flexible system that met individual needs, rather than a standard list of adjustments per illness was highly desired by students, both in this study and previous research.^6^ One participant spoke of not receiving an accommodation due to her advisor previously working with students with the same condition, who did not require that particular accommodation and another participant spoke of not knowing about an accommodation that would be useful to her, because it was on the list for a different disability. With this in mind, it also would be worth considering a DSS system of categorisation by functioning, rather than impairment.^29^

### Strengths and limitations

To the best of our knowledge, this study is the first to focus on the experiences of students with chronic illness in university education in Ireland. This was a small-scale study involving 14 participants from three universities in Ireland. There are 12 publicly funded universities in Ireland^30^ therefore the sample represents a relatively small proportion of the total. The study could have benefited from guidance from a student(s) living with chronic illness, (‘Public and Patient Involvement’ or PPI) but this was not feasible due to a lack of resourcing. Additionally, the study would have benefitted from the inclusion of a quantitative measure of health-related quality of life to give another insight into the level and types of functional limitations of participants. However, a strength of the study was that it included participants with a wide range of chronic illnesses. There were no gender differences found although fewer men participated in the study, however, this was not unexpected, since many chronic illnesses occur at higher rates in women.^31^

### Recommendations for future research, practice and policy

There is significant scope to investigate the current delivery of DSS to students living with chronic illness in universities, both in Ireland and internationally. While much research has focused on delivery of DSS generally (e.g.,^32,33^), this group, with their established unique needs, has been neglected. A useful next step would be a large-scale cross-sectional survey across multiple universities to prioritise recommendations. Peer support was identified as a potentially valuable but currently unavailable resource, indicating the need for research into its development and implementation tailored for students with chronic illnesses. Practically, universities could support this development and offer peer support groups through DSS. The findings of this study, and others,^6,15^ also imply a need for identification of the needs of teaching staff to minimise barriers to engagement for this group and ensure the delivery of inclusive education. Developing solutions to specific support needs, such as hybrid delivery of teaching and campus peer support groups, combined with a more flexible and tailored offering from DSS, could form the basis of an effective intervention to support these students. Public policy should support the implementation and funding of such initiatives to ensure inclusive and supportive educational environments.

## Conclusion

This study highlights the many challenges faced by students with chronic illnesses in university education, such as inadequate support from DSS, lack of awareness and training among teaching staff, and barriers in teaching and assessment methods. However, it also highlights areas for improvement, including establishment of peer support programs, enhanced staff training and flexible teaching methods. By implementing the recommended changes, collaborating with relevant community organisations, and receiving policy support, universities can create a more inclusive and supportive environment, ensuring that all students, regardless of their health status, have equal opportunities for success. Further research into the delivery of DSS services, the needs of teaching staff, and tailored interventions will be essential in promoting the well-being and academic success of students living with chronic illness and ensuring they don’t have to miss out on the full “college experience”.

## Supplemental Material

sj-docx-1-chi-10.1177_17423953241282246 - Supplemental material for Experiences of students with chronic illness in university education in Ireland Supplemental material, sj-docx-1-chi-10.1177_17423953241282246 for Experiences of students with chronic illness in university education in Ireland by Olga Doris and Eimear C Morrissey in Chronic Illness
