# Supplementary material for: Experiences of students with chronic illness in university education in Ireland
Source: Chronic Illn. 2024 Sep 5;21(2):277–90. doi: 10.1177/17423953241282246 (PMC12171034; doi:10.1177/17423953241282246)
Supplement: sj-docx-1-chi-10.1177_17423953241282246 - Supplemental material for Experiences of students with chronic illness in university education in Ireland [file sj-docx-1-chi-10.1177_17423953241282246.docx]

**Supplementary information**

*Interview guide*

Before commencing, the participant will be reminded of the purpose of the research and what it will involve (as per the information sheet) and that they can withdraw at any time.

• To begin, can you tell me about your illness? How long since you were diagnosed? What it is like for you? How does it affect your life generally?

• How does your illness affect your student life? What kind of barriers do you face due to your illness that affect your ability to participate fully in college life, both academically and socially?

• Can you tell me about how you manage your illness in college? What facilitates you? What strategies do you use?

• Have you registered with your college disability support services? What influenced your decision to register or not register? If registered, how suitable are the supports you receive for your particular needs?

• Have you disclosed your illness to other college staff (e.g. lecturers)? To other students? What influenced your decision to disclose or not disclose? Can you tell me about your experience of disclosing your illness?

• Can you tell me about any other supports or connections you have (e.g. other students with chronic illness, support groups)? How do these help you?

• Do you have any thoughts about how your college could better support students with chronic illnesses?

• Is there anything else you would like to add that might be helpful for this research? Do you have any questions for me?

When the interview has concluded, the participant will be debriefed. They will be provided with contact information for their university’s disability support service, counselling service and any other relevant supports. They will be advised to contact these services or their GP if they experience any negative psychological symptoms as a result of their participation in the study.

*COREQ checklist*

Developed from:

Tong A, Sainsbury P, Craig J. Consolidated criteria for reporting qualitative research (COREQ): a 32-item checklist for interviews and focus groups. *International Journal for Quality in Health Care*. 2007. Volume 19, Number 6: pp. 349 – 357.

| **No. Item** | **Guide questions/description** | **Reported on Page #** |
| --- | --- | --- |
| **Domain 1: Research team and reﬂexivity** |  |  |
| *Personal Characteristics* |  |  |
| 1. Inter viewer/facilitator | Which author/s conducted the interview or focus group? | 3 |
| 2. Credentials | What were the researcher’s credentials? E.g. PhD, MD | 3 |
| 3. Occupation | What was their occupation at the time of the study? | 3 |
| 4. Gender | Was the researcher male or female? | 3 |
| 5. Experience and training | What experience or training did the researcher have? | 3 |
| *Relationship with participants* |  |  |
| 6. Relationship established | Was a relationship established prior to study commencement? | 3 |
| 7. Participant knowledge of the interviewer | What did the participants know about the researcher? e.g. personal goals, reasons for doing the research. | 3**.** |
| 8. Interviewer characteristics | What characteristics were reported about the inter viewer/facilitator? e.g. Bias, assumptions, reasons and interests in the research topic | 3 |
| **Domain 2: study design** |  |  |
| *Theoretical framework* |  |  |
| 9. Methodological orientation and Theory | What methodological orientation was stated to underpin the study? e.g. grounded theory, discourse analysis, ethnography, phenomenology, content analysis | 3 |
| *Participant selection* |  |  |
| 10. Sampling | How were participants selected? e.g. purposive, convenience, consecutive, snowball | 3 |
| 11. Method of approach | How were participants approached? e.g. face-to-face, telephone, mail, email | 3 |
| 12. Sample size | How many participants were in the study? | 3 |
| 13. Non-participation | How many people refused to participate or dropped out? Reasons? | n/a |
| *Setting* |  |  |
| 14. Setting of data collection | Where was the data collected? e.g. home, clinic, workplace | 3 |
| 15. Presence of non-participants | Was anyone else present besides the participants and researchers? | 3 |
| 16. Description of sample | What are the important characteristics of the sample? e.g. demographic data, date | 5-6 (Table 1) |
| *Data collection* |  |  |
| 17. Interview guide | Were questions, prompts, guides provided by the authors? Was it pilot tested? | 3 |
| 18. Repeat interviews | Were repeat inter views carried out? If yes, how many? | n/a |
| 19. Audio/visual recording | Did the research use audio or visual recording to collect the data? | 3 |
| 20. Field notes | Were ﬁeld notes made during and/or after the interview or focus group? | n/a |
| 21. Duration | What was the duration of the inter views or focus group? | 3 |
| 22. Data saturation | Was data saturation discussed? | 3 |
| 23. Transcripts returned | Were transcripts returned to participants for comment and/or correction? | 3 |
| **Domain 3: analysis and ﬁndings** |  |  |
| *Data analysis* |  |  |
| 24. Number of data coders | How many data coders coded the data? | 4 |
| 25. Description of the coding tree | Did authors provide a description of the coding tree? | n/a |
| 26. Derivation of themes | Were themes identiﬁed in advance or derived from the data? | 4 |
| 27. Software | What software, if applicable, was used to manage the data? | 3 |
| 28. Participant checking | Did participants provide feedback on the ﬁndings? | 4 |
| *Reporting* |  |  |
| 29. Quotations presented | Were participant quotations presented to illustrate the themes/ﬁndings? Was each quotation identiﬁed? e.g. participant number | 7-13 |
| 30. Data and ﬁndings consistent | Was there consistency between the data presented and the ﬁndings? | 7-13 |
| 31. Clarity of major themes | Were major themes clearly presented in the ﬁndings? | 7-13 |
| 32. Clarity of minor themes | Is there a description of diverse cases or discussion of minor themes? | 7-13 |
